# Thalamic atrophy in frontotemporal dementia — Not just a *C9orf72* problem

**DOI:** 10.1016/j.nicl.2018.02.019

**Published:** 2018-02-23

**Authors:** Martina Bocchetta, Elizabeth Gordon, M. Jorge Cardoso, Marc Modat, Sebastien Ourselin, Jason D. Warren, Jonathan D. Rohrer

**Affiliations:** a Dementia Research Centre, Department of Neurodegenerative Disease, Institute of Neurology, University College London, London, United Kingdom; b Translational Imaging Group, Centre for Medical Image Computing, University College London, London, United Kingdom

**Keywords:** Frontotemporal dementia, MRI, Thalamus

## Abstract

**Background:**

Frontotemporal dementia (FTD) is a heterogeneous neurodegenerative disorder associated with frontal and temporal atrophy. Subcortical involvement has been described as well, with early thalamic atrophy most commonly associated with the *C9orf72* expansion. However thalamic involvement has not been comprehensively investigated across the FTD spectrum.

**Methods:**

We investigated thalamic volumes in a sample of 341 FTD patients (age: mean(standard deviation) 64.2(8.5) years; disease duration: 4.6(2.7) years) compared with 99 age-matched controls (age: 61.9(11.4) years). We performed a parcellation of T1 MRIs using an atlas propagation and label fusion approach to extract left and right thalamus volumes, which were corrected for total intracranial volumes. We assessed subgroups stratified by clinical diagnosis (141 behavioural variant FTD (bvFTD), 76 semantic dementia (SD), 103 progressive nonfluent aphasia (PNFA), 7 with associated motor neurone disease (FTD-MND) and 14 primary progressive aphasia not otherwise specified (PPA-NOS), genetic diagnosis (24 with *MAPT*, 24 with *C9orf72*, and 15 with *GRN* mutations), and pathological diagnosis (40 tauopathy, 61 TDP-43opathy, 3 FUSopathy). We assessed the diagnostic accuracy based on thalamic volume.

**Results:**

Overall, FTD patients had smaller thalami than controls (8% difference in volume, p < 0.0005, ANCOVA). Stratifying by genetics, *C9orf72* group had the smallest thalami (14% difference from controls, p < 0.0005). However, the thalami were also smaller than controls in the other genetic groups: *GRN* and *MAPT* groups showed a difference of 11% and 9% respectively (p < 0.0005). ROC analysis showed a relatively poor ability to separate *C9orf72* from *MAPT* (AUC = 0.651, p = 0.073) and from *GRN* cases (AUC = 0.644, p = 0.133) using thalamic volume. All clinical subtypes had significantly smaller thalami than controls (p < 0.0005), with the FTD-MND group having the smallest (15%), followed by bvFTD (9%), PNFA (8%), PPA-NOS (7%), and lastly SD (5%). In the pathological groups, the TDP-43opathies had an 11% difference from controls, and tauopathies 9%, while the FUSopathies showed only 2% of difference from controls (p < 0.0005). *GRN*, PPA-NOS and SD were the subgroups showing the highest asymmetry in volumes.

**Conclusions:**

The thalamus was most affected in *C9orf72* genetically, TDP-43opathies pathologically and FTD-MND clinically. However, thalamic atrophy is a common feature across all FTD groups.

## Highlights

• Thalamic atrophy is a common feature in frontemporal dementia.
• The thalamus was most affected in *C9orf72*, TDP-43opathies and FTD-MND.
• Thalamic volumes cannot accurately discriminate different forms of FTD.
• *GRN*, PPA-NOS and SD were the subgroups with the highest asymmetry in volumes.

## 1 Introduction

Frontotemporal dementia (FTD) is a clinically, pathologically and genetically heterogeneous neurodegenerative disorder, associated with frontal and temporal atrophy. Subcortical involvement has been found in a number of studies (Rohrer et al., 2010a; Whitwell et al., 2012; Seeley et al., 2008; Schroeter et al., 2007), with thalamic atrophy most commonly described in association with the *C9orf72* expansion (Whitwell et al., 2012; Sha et al., 2012; Lee et al., 2014; Mahoney et al., 2012), even at the presymptomatic stage (Rohrer et al., 2015; Lee et al., 2016). Other studies have reported thalamic atrophy in FTD patients (Cardenas et al., 2007; Chow et al., 2008; Garibotto et al., 2011; Hornberger et al., 2012), and in particular in those with TDP-43 pathology (Rohrer et al., 2010b), although a recent voxel-based morphometry study found thalamic involvement in both TDP-43 and tau-associated FTD cases (Harper et al., 2017). Neuropathologically, one study described a thalamic volume loss of 28–37% in FTD (Mann and South, 1993), although a more recent study only found significant thalamic atrophy in *C9orf72* cases, and not in sporadic cases with TDP-43 pathology (Yang et al., 2017). Despite these studies, it remains unclear whether and to what extent the thalamus is impaired in the other genetic forms of FTD, or across the different clinical and pathological diagnoses.

The thalamus is an important hub within many networks in the brain as it integrates somatosensory, motor, visual and auditory information through reciprocal connections with the cortex. The thalamus is composed of 50–60 different subnuclei and each nucleus has a distinct pattern of cortical and subcortical connectivity (Herrero et al., 2002). The dorsomedial and anteroventral nuclei are part of the dorsolateral prefrontal circuit, related to executive functions and motor programming, and also part of the lateral orbitofrontal circuit, related to personality and mood regulation. Being such a relevant brain structure interconnected to virtually all brain regions, the thalamus is likely to be a key structure involved in FTD. We therefore aimed to investigate thalamic involvement in a large cohort of patients across the whole FTD spectrum, including those with genetic and pathological confirmation.

## 2 Methods

We reviewed the UCL Dementia Research Centre FTD MRI database to identify 341 patients with a usable T1-weighted magnetic resonance (MR) scan and with a diagnosis of behavioural variant FTD (bvFTD) (Rascovsky et al., 2011), semantic dementia (SD), progressive nonfluent aphasia (PNFA) (Gorno-Tempini et al., 2011), FTD with associated motor neurone disease (FTD-MND), or a primary progressive aphasia not otherwise specified (PPA-NOS) (Harris et al., 2013). 99 cognitively normal subjects, with a similar age to the patients and with a usable T1-weighted MRI, were identified as controls. The study was approved by the local ethics committee and written informed consent was obtained from all participants.

MRIs were acquired from 1992 to 2017 with three different manufacturer scanners: 216 on a 1.5T Signa MRI scanner (GE Medical systems, Milwaukee, WI), 188 on a 3T Trio MRI scanner (Siemens, Erlangen, Germany), and 36 on a 3T Prisma MRI scanner (Siemens, Erlangen, Germany). When more than one MRI per participant was available, we selected the MRI closest to symptom onset. We reviewed the MRIs to make sure we excluded individuals with moderate to severe vascular disease or other brain lesions such as tumours.

For 54 patients, *post-mortem* confirmation of the underlying neuropathology was available: pathological examination of brain tissue was carried out according to standard histopathological methods at the Queen Square Brain Bank for Neurological Disorders, UCL Institute of Neurology. 67 patients were carriers of a mutation in one of the FTD-linked genes: microtubule-associated protein tau (*MAPT*) (Hutton et al., 1998; Ghetti et al., 2015), progranulin (*GRN*) (Baker et al., 2006; Cruts et al., 2006), chromosome 9 open reading frame 72 (*C9orf72*) (DeJesus-Hernandez et al., 2011; Renton et al., 2011), TANK-binding kinase 1 (*TBK1*) (Freischmidt et al., 2015; Gijselinck et al., 2015; Le Ber et al., 2015; Pottier et al., 2015), and sequestosome 1 (*SQSTM1*) (Rubino et al., 2012; Le Ber et al., 2013; Miller et al., 2015). We divided the patient group based on their clinical diagnosis (141 bvFTD, 76 SD, 103 PNFA, 7 FTD-MND, 14 PPA-NOS), their genetic diagnosis (24 *MAPT*, 24 *C9orf72*, 15 *GRN*), and their pathological diagnosis (40 tauopathy, 61 TDP-43opathy, 3 FUSopathy). Within the tauopathy group, we included individuals who had tau pathology at *post-mortem* or were *MAPT* mutation carriers, while within the TDP-43opathy group, we included individuals who had definite TDP-43 pathology or were carriers of mutations in *GRN*, *C9orf72*, *TBK1* (n = 2), and dual mutations in *GRN*/*C9orf72* (n = 1) or *C9orf72*/*SQSTM1* (n = 1) (Table 1; Supplementary Table 1).

**Table 1:** Demographic and clinical variables for the FTD patients and controls, together with thalamic volumes. Values denote mean (standard deviation) or n (%).

Groups		n	Gender, male	Age at scan (years)	Disease duration (years)	Left thalamic volume (as % of TIV)	Right thalamic volume (as % of TIV)	Total thalamic volume (as % of TIV)
Controls		99	42 (42%)	61.9 (11.4)	–	0.41 (0.03)	0.40 (0.03)	0.80 (0.06)
Clinical	FTD-MND	7	4 (57%)	66.1 (3.8)	4.6 (2.4)	0.34 (0.03)	0.35 (0.03)	0.69 (0.06)
	bvFTD	141	41 (29%)	61.3 (8.3)	5.2 (3.2)	0.37 (0.03)	0.37 (0.02)	0.73 (0.05)
	PNFA	103	50 (49%)	68.3 (8.5)	4.3 (2.2)	0.36 (0.03)	0.38 (0.03)	0.74 (0.06)
	PPA-NOS	14	10 (71%)	63.9 (6.3)	3.3 (1.7)	0.36 (0.03)	0.39 (0.03)	0.75 (0.06)
	SD	76	42 (55%)	64.0 (7.4)	4.6 (2.3)	0.37 (0.03)	0.40 (0.03)	0.77 (0.05)
Genetic	*C9orf72*	24	17 (71%)	60.9 (6.9)	5.6 (3.2)	0.35 (0.03)	0.34 (0.03)	0.69 (0.06)
	*GRN*	15	7 (47%)	62.6 (6.6)	2.9 (2.7)	0.36 (0.04)	0.35 (0.02)	0.72 (0.04)
	*MAPT*	24	15 (63%)	55.4 (5.7)	5.7 (3.3)	0.37 (0.04)	0.36 (0.04)	0.73 (0.07)
Pathological	TDP-43	60	38 (63%)	63.1 (6.9)	4.6 (3.0)	0.37 (0.04)	0.35 (0.03)	0.72 (0.06)
	Tau	40	28 (70%)	58.5 (8.5)	5.1 (2.8)	0.37 (0.04)	0.36 (0.03)	0.73 (0.07)
	FUS	3	2 (67%)	43.9 (13.6)	3.3 (2.1)	0.39 (0.02)	0.40 (0.04)	0.79 (0.07)

Thalamic volumes were extracted as part of the parcellation on T1-weighted volumetric MRI scans as previously described (Rohrer et al., 2015), using an atlas propagation and label fusion strategy (Cardoso et al., 2015). Volumes are expressed as a percentage of total intracranial volume (TIV), computed with SPM12 v6470 (Statistical Parametric Mapping, Wellcome Trust Centre for Neuroimaging, London, UK) running under Matlab R2014b (Math Works, Natick, MA, USA) (Malone et al., 2015). All segmentations were visually checked for quality. Statistical analyses were performed in SPSS software (SPSS Inc., Chicago, IL, USA) v22.0, between control and patient groups, using the ANCOVA test adjusting for scanner type, gender and age. When comparing between different patient subgroups, we also adjusted for disease duration. Results were corrected for multiple comparisons (Bonferroni's correction), p < 0.008 for the clinical groups, p < 0.013 for the genetic and pathological groups. To assess the accuracy of the thalamic volume in discriminating between different diagnoses, we performed a Receiver Operating Characteristic (ROC) analysis. We also investigated asymmetry by calculating an Asymmetry Index (AI), defined as the absolute difference between the left and right thalamic volumes in relation to the total bilateral volume: |(Left − Right)|/(Left + Right).

## 3 Results

Sociodemographic and clinical data are reported in Table 1. The mean disease duration for the whole FTD group at the time of the scan was 4.6 years (standard deviation 2.7) with an average age at onset at 59.6 (8.6). There was no significant difference in age between FTD and controls (p = 0.067, t-test), or for scanner type (p = 0.785, Chi square test), but there were more males in the FTD group than in the control group (60% vs 42%, p = 0.002, Chi square test). Across the different clinical, genetic and pathological diagnoses, there was no difference for scanner type (p = 0.266, p = 0.508 and p = 0.390, Chi square test). There was a significant difference in disease duration across the genetic groups (p = 0.016, ANOVA), with *GRN* carriers showing the shortest duration, but no difference across the pathological nor clinical groups (p = 0.433 and p = 0.075).

Investigating the control group, there was a weak but significant negative correlation of thalamic volume with age (Spearman's rho: −0.444, p-value < 0.0005).

**Fig. 1:**
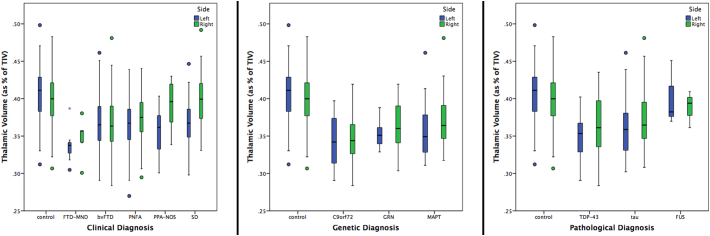
Volume of the left and right thalamus as a percentage of total intracranial volume in 341 FTD patients and 99 controls, by clinical, genetic and pathological groups.

Overall, the total FTD group had significantly smaller thalami than controls (10% and 6% difference in the left and right volume respectively, p < 0.0005, ANCOVA). All clinical subtypes showed significantly smaller thalami than controls (p < 0.0005, ANCOVA), with the FTD-MND group having the smallest (17 and 13%, left and right), followed by bvFTD (10 and 8%), PNFA (10 and 6%), PPA-NOS (12 and 2%) and lastly SD (9 and 0.1%) (Fig. 1). Comparing disease groups, FTD-MND showed significantly smaller volumes when compared to all the other clinical subgroups, except for PPA-NOS; bvFTD showed significantly smaller thalami than PNFA, PPA-NOS and SD; PNFA showed a smaller right thalamus than PPA-NOS and bilaterally smaller volumes than SD; while PPA-NOS showed bilaterally smaller thalami than SD (Fig. 1, Table 2).

**Table 2:** Volumetric comparisons and diagnostic accuracy between the different clinical, genetic and pathological subgroups for the right, left and total thalamic volume. Volumetric comparisons are adjusted for age, gender, scanner type and disease duration. AUC = Area under the curve. Bold represents a significant difference between groups after correcting for multiple comparisons.

Clinical diagnosis		bvFTD				PNFA				PPA-NOS				SD			
		ANCOVA		AUC		ANCOVA		AUC		ANCOVA		AUC		ANCOVA		AUC	
		% difference	p-value	AUC (95% CI)	p-value	% difference	p-value	AUC (95% CI)	p-value	% difference	p-value	AUC (95% CI)	p-value	% difference	p-value	AUC (95% CI)	p-value
FTD-MND	Right thalamic volume (as % of TIV)	5%	0.009	0.650 (0.496–0.805)	0.180	**7%**	**<0.0005**	0.773 (0.640–0.905)	0.016	11%	0.073	**0.867 (0.710–1.000)**	**0.007**	**13%**	**<0.0005**	**0.921 (0.834–1.000)**	**<0.0005**
	Left thalamic volume (as % of TIV)	8%	0.014	0.769 (0.604–0.934)	0.016	7%	0.032	0.753 (0.579–0.927)	0.025	5%	0.728	0.673 (0.425–0.922)	0.205	**8%**	**0.001**	**0.816 (0.629–1.000)**	**0.006**
	Thalamic volume (as % of TIV)	**6%**	**0.007**	0.718 (0.561–0.876)	0.052	**7%**	**<0.0005**	0.764 (0.609–0.920)	0.020	8%	0.296	0.827 (0.639–1.000)	0.017	**10%**	**<0.0005**	**0.883 (0.750–1.000)**	**0.001**
bvFTD	Right thalamic volume (as % of TIV)	–				**3%**	**<0.0005**	0.592 (0.520–0.663)	0.015	**6%**	**<0.0005**	0.712 (0.576–0.848)	0.009	**8%**	**<0.0005**	**0.763 (0.700–0.826)**	**<0.0005**
	Left thalamic volume (as % of TIV)					**-1%**	**0.004**	0.495 (0.422–0.568)	0.894	-3%	0.035	0.415 (0.263–0.567)	0.294	**0%**	**0.001**	0.520 (0.442–0.599)	0.620
	Thalamic volume (as % of TIV)					**1%**	**<0.0005**	0.549 (0.476–0.622)	0.194	**2%**	**0.007**	0.582 (0.426–0.738)	0.312	**4%**	**<0.0005**	**0.666 (0.593–0.739)**	**<0.0005**
PNFA	Right thalamic volume (as % of TIV)	–				–				**4%**	**<0.0005**	0.637 (0.480–0.794)	0.096	**6%**	**<0.0005**	**0.697 (0.620–0.774)**	**<0.0005**
	Left thalamic volume (as % of TIV)									-2%	0.099	0.417 (0.265–0.570)	0.318	**1%**	**0.002**	0.521 (0.436–0.606)	0.624
	Thalamic volume (as % of TIV)									**1%**	**0.002**	0.537 (0.374–0.700)	0.656	**3%**	**<0.0005**	**0.620 (0.538–0.702)**	**0.006**
PPA-NOS	Right thalamic volume (as % of TIV)	–				–				–				**2%**	**<0.0005**	0.555 (0.388–0.721)	0.518
	Left thalamic volume (as % of TIV)													**3%**	**0.003**	0.611 (0.442–0.780)	0.189
	Thalamic Volume (as % of TIV)													**3%**	**0.001**	0.582 (0.415–0.748)	0.333


Genetic diagnosis		*GRN*				*MAPT*			
		ANCOVA		AUC		ANCOVA		AUC	
		% difference	p-value	AUC (95% CI)	p-value	% difference	p-value	AUC (95% CI)	p-value
*C9orf72*	Right thalamic volume (as % of TIV)	4%	0.302	0.617 (0.430–0.803)	0.225	7%	0.071	0.679 (0.527–0.831)	0.034
	Left thalamic volume (as % of TIV)	3%	0.879	0.594 (0.416–0.772)	0.326	4%	0.198	0.606 (0.444–0.767)	0.208
	Thalamic volume (as % of TIV)	4%	0.678	0.644 (0.471–0.818)	0.133	5%	0.113	0.651 (0.496–0.806)	0.073
*GRN*	Right thalamic Volume (as % of TIV)	–				2%	0.050	0.547 (0.355–0.740)	0.624
	Left thalamic volume (as % of TIV)					1%	0.183	0.514 (0.332–0.696)	0.885
	Thalamic volume (as % of TIV)					2%	0.036	0.519 (0.337–0.702)	0.840


Pathological Diagnosis		tau				FUS			
		ANCOVA		AUC		ANCOVA		AUC	
		% difference	p-value	AUC (95% CI)	p-value	% difference	p-value	AUC (95% CI)	p-value
TDP-43	Right thalamic volume (as % of TIV)	2%	0.051	0.542 (0.429–0.655)	0.479	6%	0.059	0.678 (0.500–0.856)	0.302
	Left thalamic volume (as % of TIV)	2%	0.290	0.550 (0.434–0.666)	0.393	13%	0.119	0.869 (0.736–1.000)	0.032
	Thalamic volume (as % of TIV)	2%	0.083	0.546 (0.432–0.661)	0.433	9%	0.095	0.803 (0.592–1.000)	0.078
tau	Right thalamic Volume (as % of TIV)	–				4%	0.068	0.658 (0.430–0.886)	0.365
	Left thalamic volume (as % of TIV)					**11%**	**0.011**	0.825 (0.664–0.986)	0.063
	Thalamic volume (as % of TIV)					7%	0.014	0.767 (0.535–0.998)	0.127

Stratifying by genetics, *C9orf72* group had the smallest thalami (left: 15 and right: 13% difference from controls, p < 0.0005). However, the thalami were also smaller than controls in the other genetic groups: *GRN* (13 and 9%, p < 0.0005) and *MAPT* (12 and 7%, p < 0.0005) groups. There were no significant differences between the disease groups. Note that excluding the genetic cases and analyzing the sporadic cases alone (n = 274) also showed a similar pattern of smaller thalami than controls (4% difference on the left, 9% on the right, and 7% in total, p < 0.0005 ANCOVA - Supplementary Table 2 for more detailed analysis).

In the pathological groups, the TDP-43opathies had a 14% (left) and 8% (right) difference from controls (p < 0.0005), and tauopathies 12 and 6% (p < 0.0005), while the FUSopathies showed the smallest difference from controls (1 and 3%, p < 0.0005). Comparing disease groups, only the tau group showed a smaller (left) thalamic volume than the FUS group (p = 0.011) (Table 2).

Among the clinical groups, the ROC analysis showed the highest diagnostic accuracy between FTD-MND and SD (right thalamus, AUC = 0.921, p < 0.0005) (Table 2). In the genetic groups the ROC analysis showed a poor ability to separate *C9orf72* from *MAPT*, with the highest AUC value for the right thalamus (AUC = 0.679, p = 0.034) and from *GRN* cases (AUC = 0.644, p = 0.133 for the sum of right and left) (Table 2). For the pathological groups, the highest accuracy was when differentiating between TDP-43 and tau for the left thalamus volume (AUC = 0.869, p-value = 0.032).

**Fig. 2:**
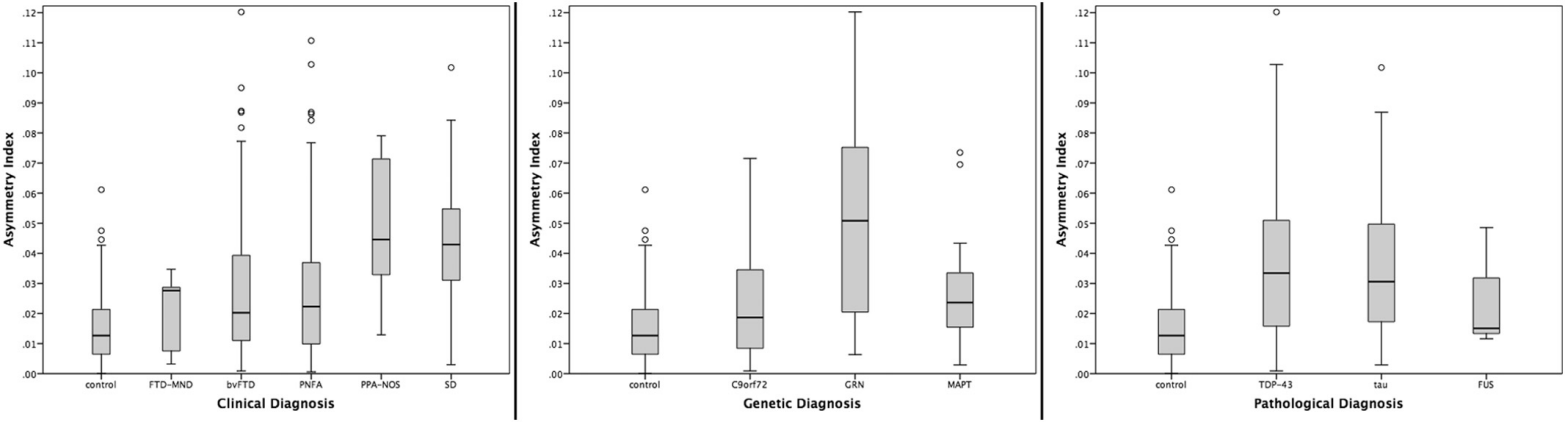
Asymmetry Index for the thalamus in 341 FTD patients and 99 controls, by clinical, genetic and pathological groups.

When investigating the asymmetry of the thalamus, the FTD group as a whole was significantly more asymmetric than controls (0.032 (0.024) versus 0.015 (0.012), <0.00005, ANCOVA). However, consistent with previous findings, the controls had a non-zero asymmetry index. PPA-NOS and SD were the most asymmetric clinical groups, and *GRN* the most asymmetric among the genetic groups (Fig. 2 and Table 3). FUS, FTD-MND and *C9orf72* were the only groups not showing significant asymmetry.

**Table 3:** Asymmetry values and comparisons between the different clinical, genetic and pathological subgroups for the right, left and total thalamic volume. Analyses are adjusted for age, gender, scanner type and disease duration.

	Mean (SD)	p-value				
Clinical diagnosis		FTD-MND	bvFTD	PNFA	PPA-NOS	SD
Control	0.015 (0.012)	0.289	<0.00005	<0.00005	<0.00005	<0.00005
FTD-MND	0.020 (0.013)	–	0.887	0.313	0.015	<0.00005
bvFTD	0.028 (0.023)	–	–	0.669	0.061	<0.00005
PNFA	0.028 (0.024)	–	–	–	0.020	<0.00005
PPA-NOS	0.048 (0.023)	–	–	–	–	<0.00005
SD	0.042 (0.020)	–	–	–	–	–


Genetic diagnosis		*C9orf72*	*GRN*	*MAPT*
Control	0.015 (0.012)	0.124	<0.00005	<0.00005
*C9orf72*	0.022 (0.017)	–	<0.00005	0.879
*GRN*	0.051 (0.036)	–	–	0.007
*MAPT*	0.027 (0.017)	–	–	–


Pathological diagnosis		TDP-43	Tau	FUS
Control	0.015 (0.012)	<0.00005	<0.00005	0.151
TDP-43	0.037 (0.026)	–	<0.00005	0.002
Tau	0.036 (0.024)	–	–	0.117
FUS	0.025 (0.020)	–	–	–

## 4 Discussion

Using an automated and robust segmentation method to segment the thalamus in a large cohort of FTD patients, we demonstrated that thalamic volumes were lower than in controls in all clinical, genetic, and pathological FTD groups except those with FUSopathies, and that FTD-MND, *C9orf72*, and TDP-43 were the subgroups for which the thalamus was particularly affected. Our results support the existing literature on *C9orf72* (Whitwell et al., 2012; Sha et al., 2012; Lee et al., 2014; Mahoney et al., 2012), and on TDP-43 pathology (Rohrer et al., 2010b). However, these results show that thalamic atrophy is not just a characteristic of *C9orf72* (Yang et al., 2017), but of the whole FTD spectrum, and it is not possible to accurately discriminate among different forms of FTD based on thalamic volumes.

The overall difference in thalamic volumes compared with controls was smaller than in the two neuropathological studies of thalamus atrophy that have been performed [28–37% in Mann and South, 1993; 46–49% in *C9orf72* carriers and ~25% in sporadic FTD due to TDP-43 pathology in Yang et al., 2017], but our measurements were done *in vivo* on MRI and as close as possible to the diagnosis. They are likely to be lower therefore than any pathological studies where the disease will be at a more severe stage.

The most significant clinical, genetic and pathological groups overlap in our study, as FTD-MND is usually a TDP-43opathy and is commonly associated with a *C9orf72* mutation. Four out of 7 of the FTD-MND cases here had either a single *C9orf72* mutation (2 cases) or a dual mutation of *C9orf72* with another gene (2 cases). However it is clear that lower thalamic volumes are not just driven by the *C9orf72* status with overlap of values with other mutations and pathologies.

Previous studies have shown involvement of the thalamus in patients with ALS without FTD (e.g. in *C9orf72*-ALS: Bede et al., 2013) and it will be useful to study the FTD/MND continuum further in larger cohorts, given the relatively limited size of the FTD-MND group in this study.

Of all the groups, the FUSopathies seems to have the most intact thalamus, consistent with previous imaging studies (Rohrer et al., 2011). However, this is a rare pathological cause of FTD and there were only 3 patients in this group – larger studies will be required to investigate this further.

Clinically, while the combined right and left thalamic volume was highest for PPA-NOS and SD, this was driven by the asymmetrical nature of the disease, with much lower volumes of the left compared with the right thalamus (and the highest asymmetry index). The *GRN* mutation group also showed asymmetry consistent with previous studies showing that this is generally a very asymmetrical disease (Rohrer et al., 2010a).

The study included a large control group of 99 healthy individuals. Our study supported two key findings in the literature of firstly, a correlation between age and thalamic volume [previous studies showing an R^2^ ranging from 0.31 to 0.60: Sullivan et al., 2004; Hughes et al., 2012], and evidence for asymmetry of thalamic volume [e.g. in a study of over 15,800 people by Guadalupe et al., 2016, AI was 0.021, comparable with the finding here of 0.015].

The thalamus is a key hub in several brain networks and it is therefore not surprising that it is involved in all the different forms of FTD. We only investigated the whole thalamus volume here, but each thalamic nucleus has a distinct pattern of cortical and subcortical connectivity. Future studies, including functional and structural connectivity MR analyses of the thalamus, will be needed to investigate the different subnuclei and their connections in order to better understand the role of this key structure in each of the different forms of FTD. Furthermore, improved understanding of the role of the thalamus in different cognitive and behavioural functions may allow better stratification clinically of the FTD syndromes (e.g. those that have abnormal pain and temperature processing (Fletcher et al., 2015)) and therefore clearer correlation with specific subnuclei. Lastly, the findings of this study suggest that thalamic atrophy may be a useful volumetric imaging biomarker in future disease modifying therapy trials as it is easily measured and universal to all FTD subtypes.

## Appendix A Supplementary data

Supplementary tables Image 1
