# Supplementary material for: Thalamic atrophy in frontotemporal dementia — Not just a C9orf72 problem
Source: Neuroimage Clin. 2018 Feb 23;18:675–81. doi: 10.1016/j.nicl.2018.02.019 (PMC5988457; doi:10.1016/j.nicl.2018.02.019)
Supplement: Supplementary file 1 — Supplementary tables [file mmc1.docx]

**Supplementary Table 1. Number of mutation carriers (1A) and pathological diagnosis (1B) for each clinical diagnosis group.**

**1A**

|  | | **Genetic Diagnosis** | | | | | |
| --- | --- | --- | --- | --- | --- | --- | --- |
|  |  | ***C9orf72*** | ***GRN*** | ***MAPT*** | ***GRN+C9orf72*** | ***C9orf72+SQSTM1*** | Total |
| **Clinical Diagnosis** | **FTD-MND** | 2 | 0 | 0 | 1 | 1 | 4 |
|  | **bvFTD** | 20 | 9 | 23 | 0 | 0 | 52 |
|  | **PNFA** | 2 | 4 | 1 | 0 | 0 | 7 |
|  | **PPA-NOS** | 0 | 2 | 0 | 0 | 0 | 2 |
|  | **SD** | 0 | 0 | 0 | 0 | 0 | 0 |
|  | Total | 24 | 15 | 24 | 1 | 1 | 65 |

**1B**

|  | | **Pathological Diagnosis** | | | |
| --- | --- | --- | --- | --- | --- |
|  |  | **TDP-43** | **tau** | **FUS** | Total |
| **Clinical Diagnosis** | **FTD-MND** | 4 | 0 | 0 | 4 |
|  | **bvFTD** | 31 | 31 | 3 | 65 |
|  | **PNFA** | 8 | 7 | 0 | 15 |
|  | **PPA-NOS** | 2 | 0 | 0 | 2 |
|  | **SD** | 16 | 2 | 0 | 18 |
|  | Total | 61 | 40 | 3 | 104 |

**Supplementary Table 2. Volumetric comparisons between the different clinical subgroups in the sporadic cohort for the right, left and total thalamic volume.** Volumetric comparisons are adjusted for age, gender and scanner type, and also for disease duration when comparing between FTD groups. Bold represents a significant difference between groups after correcting for multiple comparisons.

| **Clinical Diagnosis (sporadic only)** | | **Controls** | | **bvFTD** | | **PNFA** | | **PPA-NOS** | | **SD** | |
| --- | --- | --- | --- | --- | --- | --- | --- | --- | --- | --- | --- |
|  |  | **ANCOVA** | | **ANCOVA** | | **ANCOVA** | | **ANCOVA** | | **ANCOVA** | |
|  |  | % difference | p-value | % difference | p-value | % difference | p-value | % difference | p-value | % difference | p-value |
| **FTD-MND** | Right Thalamic Volume (as % of TIV) | **10%** | **<0.0005** | 4% | 0.030 | **5%** | **<0.0005** | 8% | 0.199 | **10%** | **<0.0005** |
|  | Left Thalamic Volume (as % of TIV) | **17%** | **<0.0005** | **10%** | **0.005** | 8% | 0.016 | 5% | 0.522 | **8%** | **0.003** |
|  | Thalamic Volume (as % of TIV) | **14%** | **<0.0005** | **7%** | **0.005** | **7%** | **<0.0005** | 7% | 0.326 | **9%** | **<0.0005** |
| **bvFTD** | Right Thalamic Volume (as % of TIV) | **7%** | **<0.0005** | -- | | **2%** | **<0.0005** | **4%** | **0.005** | **7%** | **<0.0005** |
|  | Left Thalamic Volume (as % of TIV) | **8%** | **<0.0005** |  |  | **-2%** | **<0.0005** | **-5%** | **0.003** | **-2%** | **<0.0005** |
|  | Thalamic Volume (as % of TIV) | **7%** | **<0.0005** |  |  | **0%** | **<0.0005** | **0%** | **0.006** | **3%** | **<0.0005** |
| **PNFA** | Right Thalamic Volume (as % of TIV) | **5%** | **<0.0005** | -- | | -- | | **3%** | **<0.0005** | **5%** | **<0.0005** |
|  | Left Thalamic Volume (as % of TIV) | **10%** | **<0.0005** |  |  |  |  | -3% | 0.020 | **0%** | **<0.0005** |
|  | Thalamic Volume (as % of TIV) | **8%** | **<0.0005** |  |  |  |  | **0%** | **0.001** | **3%** | **<0.0005** |
| **PPA-NOS** | Right Thalamic Volume (as % of TIV) | **3%** | **<0.0005** | -- | | -- | | -- | | **3%** | **<0.0005** |
|  | Left Thalamic Volume (as % of TIV) | **12%** | **<0.0005** |  |  |  |  |  |  | **3%** | **0.003** |
|  | Thalamic Volume (as % of TIV) | **8%** | **<0.0005** |  |  |  |  |  |  | **3%** | **0.001** |
| **SD** | Right Thalamic Volume (as % of TIV) | **0%** | **<0.0005** | -- | | -- | | -- | | -- | |
|  | Left Thalamic Volume (as % of TIV) | **9%** | **<0.0005** |  |  |  |  |  |  |  |  |
|  | Thalamic Volume (as % of TIV) | **5%** | **<0.0005** |  |  |  |  |  |  |  |  |
